# Supplementary material for: Cis-regulatory differences in isoform expression associate with life history strategy variation in Atlantic salmon
Source: PLoS Genet. 2020 Sep 30;16(9):e1009055. doi: 10.1371/journal.pgen.1009055 (PMC7549781; doi:10.1371/journal.pgen.1009055)
Supplement: S1 Table — (DOCX) [file pgen.1009055.s011.docx]

Table S 1

| *Vgll3* 5’ UTR Forward Primer | *Vgll3* 5’ UTR Reverse Primer | *Vgll3* 5’ UTR *Late* probe | *Vgll3* 5’ UTR *Early* probe |
| --- | --- | --- | --- |
| GTCGTCATACTTTCACCAGAACACA | TGTAAACACCCAGCTTCTTCGAA | AAAAGTTGTAGGACGTTTC | AAAAGTTGTAGAACGTTTC |

| *Vgll3* Exon 2 Forward Primer | *Vgll3* Exon 2 Reverse Primer | *Vgll3* Exon 2 *Early* probe | *Vgll3* Exon 2 *Late* probe |
| --- | --- | --- | --- |
| GAAGCTGGGTGTTTACAGTAGGAT | TGTCTCCGCCCTGGAAAC | TGCTGCTCCATGCTGT | TGCTGCTCCGTGCTGT |

| *Vgll3* Exon 3 Forward Primer | *Vgll3* Exon 3 Reverse Primer | *Vgll3* Exon 3 *Early* probe | *Vgll3* Exon 3 *Late* probe |
| --- | --- | --- | --- |
| AGCCCAGGGATACACAGTGA | GTGGGCCAGGCTGAGG | CCACCTCTGTGTTCACA | CCACCTCTGTCTTCACA |

| *Amh* Forward Primer | *Amh* Reverse Primer | *Amh* probe |
| --- | --- | --- |
| ATGAAAGAGTGACAGAAAAGCTGAGT | TGCAGCTCACACAGAAACGT | CCCTCCCCTGCTCCC |

| *Igf3* Forward Primer | *Igf3* Reverse Primer | *Igf3* probe |
| --- | --- | --- |
| CAACAAACTGAGATGAACAGTCTGAAC | CAGTCGCACAGCCAAGAATAAAA | TCGGCACAAAATATCT |
