# Supplementary material for: Cis-regulatory differences in isoform expression associate with life history strategy variation in Atlantic salmon
Source: PLoS Genet. 2020 Sep 30;16(9):e1009055. doi: 10.1371/journal.pgen.1009055 (PMC7549781; doi:10.1371/journal.pgen.1009055)
Supplement: Attachment — Submitted filename: Review.pdf [file pgen.1009055.s012.pdf]

In this study, the authors investigate how genetic variation in the regulatory sequence of a gene previously identified by GWAS influences a fitness-related trait, the age at sexual maturity in Atlantic salmon males. By crossing males and females with known genotype at the *vgll3* locus (a transcription co-factor gene) and raising their progeny in a common environment, they confirmed that allelic variation at this locus was associated with the timing of male puberty. They then quantified *vgll3* mRNA levels in ten tissues at different developmental stages, showing that the expression of this gene was elevated in immature testes and decreased after sexual maturation. In addition, allelic variation at the *vgll3* locus was associated with a 20% change in mean *vgll3* mRNA level in immature testes. By quantifying expression of each *vgll3* allele in heterozygote individuals, the authors conclude that the expression difference between the two alleles is largely explained by *cis*-regulatory variation. They propose a plausible model whereby a *cis*-regulatory change in a *vgll3* enhancer would affect the average expression level of *vgll3* in immature testes, contributing to variation in male puberty timing.

By unravelling a molecular mechanism underlying variation in a key life history trait of a non-model organism, I expect this work to have a broad impact on the readership of *PLoS Genetics*. I particularly appreciated the clarity and concision of the introduction and discussion. However, I have a few concerns that I think should be addressed before publication. In my opinion, the article would benefit from a more detailed description of some of the methods used and from showing more clearly critical controls in some figures, as suggested below. This would strengthen the conclusion that *cis*-regulatory variation contributes to the expression difference observed between *vgll3* alleles.

Here is my list of concerns and suggestions ranked from highest to lowest priority:

1) In page 9, the authors wrote: "the average magnitude of the *cis*-regulatory differences observed […] was large enough to account for 62% of the average expression differences between […] homozygotes.". I do not understand how they obtained this 62% estimate. They should explain it in the Results and Methods sections, since this is critical to support the conclusion that the expression difference between *vgll3* alleles is largely *cis*-regulatory. I found this 62% estimate surprisingly high since the *L* allele only represented 53% of total *vgll3* mRNA in heterozygotes (as compared to 50% expected in absence of *cis*-regulatory effect) and the mean *vgll3* mRNA difference between homozygotes was 20%.

2) Related to the first point, in the introduction the authors proposed the hypothesis that a *cis*-regulatory change can underlie the association between *vgll3* genotype and age at maturity since the strongest association (in the previous GWAS study) mapped to a non-coding sequence 7000 bp upstream of *vgll3* coding sequence. While this seems to be the most likely explanation, previous studies in other organisms showed that genetic variation in an enhancer can have *trans*-regulatory effect on expression of nearby genes (Tian et al. PLoS Genet. 2019 doi: 10.1371/journal.pgen.1008152; Bateman et al. Genetics 2012 doi: 10.1534/genetics.112.140954). The authors could mention this alternative hypothesis in the introduction or in the discussion.

3) The null hypothesis for testing allele specific expression in Figure 3B was obtained from heterozygote DNA. This is an essential control to assess the statistical significance of the *cis*-regulatory effect observed, and I am glad the authors did it. However, they do not provide much information about this control. How many replicate samples were used to obtain the dashed line on Figure 3B? How were these data used to compute the P-values shown on Figure 3B? I would also suggest mentioning in the main text why this control was performed,

which I suppose was to account for biases in the specificity of the PCR primers. This would more convincingly convey that the 53% deviation (weak but significant) toward the *L* allele is not an experimental artifact.

4) For a non-specialist in fish biology, the methods used to assess sexual maturity seem a bit subjective (Page 13: "We […] assessed the maturity status of males by manually testing for the presence of running milt"; Page 14: "We classified each male fish visually as immature (not enlarged, transparent color gonads), pubertal (slightly enlarged, transparent or white color gonads) or mature (enlarged white color gonads)"). I am wondering if it would be possible to collect more quantitative data about this central phenotype, for instance by weighing the dissected gonads. At the very least, it would be helpful for non-specialist readers to mention in the text that the age of sexual maturity can be difficult to measure accurately. I guess this is why the authors used quantitative measures such as *amh/igf3* ratio, which could be stated more clearly.

5) In page 5, the authors mentioned a 9% vs 0% difference in mean maturation probability and a 10 times higher frequency of maturation in individuals with *vgll3*EE* genotypes compared to individuals with *vgll3*LL* genotypes. I do not understand the distinction between "mean maturation probability" and "frequency of maturation", and how these values were obtained. Related to this point, it is unclear how the error bars were calculated on Figure 1A. It would also be nice to mention the number of individuals in the legend. For clarity, the legend of Figure 1B should mention the type of model used (generalized linear mixed model).

6) The experimental design could be explained with a bit more details to be more accessible for non-specialists. For instance, I suggest to move Figure S1 as a panel in Figure 1. If I understood well, each of 16 males were crossed to 2 females and each of 16 females were crossed to 2 males to produce a total of 32 families. Were they all crossed simultaneously or consecutively? How were the families with common parents distinguished? The authors wrote on Page 13 that each tank included on average six individuals per family, but it is unclear whether each tank included individuals from all families or not. Also, I assume that a given number of individuals from each family was randomly selected for each experiment, but this process is not clearly explained. For instance, did the experimenters know the *vgll3* genotype when assessing maturity or was it done in a blind way?

7) In Figure 2, all expression measures were obtained using a digital PCR approach. Since this approach is more sensitive than standard qPCR, the authors should explicitly mention that they used this approach in the main text as well as in Figure 2 legend. They mentioned in the Methods that they verified the specificity of all TaqMan assays with genomic and synthetic DNA templates, but did not provide data for these important controls. I suggest that they include a supplementary figure showing the specificity of the primers used in digital PCR assays.

8) In Figure 3B, the y-axis should be L / (L + E) instead of L / E. The legend mentions black and grey colors, while the color scale on the figure is dark to light green. I also suggest using different symbols to distinguish data points with P-value < 0.05 (triangles for instance).

9) In Page 3, the authors wrote: "the transcription co-factor gene *vestigial-like 3* (*vgll3*) was recently found to explain nearly 40% of variation in sea-age at maturity". Was this

association found only in males, also in females, or regardless of sex? I think this information should be mentioned in the text.

10) There is a grammatical problem with the first sentence of the third paragraph in Page 13: “Before first feeding and transfer…”.
