# Supplementary material for: Cis-regulatory differences in isoform expression associate with life history strategy variation in Atlantic salmon
Source: PLoS Genet. 2020 Sep 30;16(9):e1009055. doi: 10.1371/journal.pgen.1009055 (PMC7549781; doi:10.1371/journal.pgen.1009055)
Supplement: Attachment — Submitted filename: verta_et_al_response_to_reviews - Copy.pdf [file pgen.1009055.s013.pdf]

UNIVERSITY OF HELSINKI
FACULTY OF BIOLOGICAL AND ENVIRONMENTAL SCIENCES

Dear Dr. Juliette de Meaux, dear reviewers,

Thank you for the many constructive comments you made on the submitted version of our manuscript. We regret the delays in returning these revisions due to Covid-19 lockdown of our university. We performed extensive additional experiments to validate our results. These experiments were clearly worthwhile, as we discovered additional exciting details into the mechanism of genotype-phenotype association between *vgll3* and age at maturity. Below we briefly explain our additional experiments and these new results, before addressing your comments on our previous version.

- We designed two new targeted expression assays for different exons of the *vgll3* transcript to validate the association between *vgll3 cis*-expression and maturation probability. Using these assays we discovered that expression differences between *vgll3* genotypes varied for different exons of the *vgll3* transcript. Our careful control experiments ruled out the possibility that the expression differences could be due to technical artefacts.
- We characterized the *vgll3* transcript structure using RACE (Rapid Amplification of cDNA Ends), hypothesizing that variation in the expression of previously uncharacterized *vgll3* isoforms (transcript variants) could explain the expression differences between exons of the *vgll3* transcript. We discovered that all genotypes expressed a mixture of at least two *vgll3* transcript isoforms, of which one was expressed from a previously uncharacterized 5' UTR within the first intron. Furthermore, using Sanger sequencing we discovered that the novel 5' UTR contained a C/T SNP that was linked to the *late*/*early vgll3* genotype.
- Analysis of heterozygote expression with a targeted expression assay for the alternative 5' UTR showed that it was preferentially expressed from the *late* allele, and that differences in exon expression levels were *cis*-regulatory as well.
- We validated these new results by analyzing a publicly available, independent RNA-seq data-set of immature testes expression. The dataset showed a consistent pattern, with the alternative 5' UTR expressed from the *late* allele and an allele-specific difference in exon 2 expression.

These new results strongly suggest that the association between *vgll3* genotype and maturation probability is driven by *cis*-regulatory difference in *vgll3* isoform expression. As such, our revised model is that the genotype-phenotype association in *vgll3* has a mechanistic

Dr. Jukka-Pekka Verta
Viikinkaari 9, 00790, Helsinki, Finland
jukka-pekka.verta@helsinki.fi
helsinki.fi/en/researchgroups/evolution-conservation-and-genomics
researchportal.helsinki.fi/en/persons/jukka-pekka-verta

basis similar to e.g. sex determination in many insect species, where phenotype differences are due to expression of alternative transcript isoforms.

We acknowledge that much more data and likely a completely new approach is needed to fully disentangle the complexity of *vgll3* isoform expression. However, we also believe we have provided exciting new evidence to firmly conclude the existence of an isoform-mediated mechanism in maturation timing control in Atlantic salmon and due to the competitive nature of this field, it would be timely to publish the current results.

Below you will find our point by point responses to the previous comments.

Sincerely,

Jukka-Pekka Verta
Post-doctoral researcher
Organismal and Evolutionary Biology Research Programme
University of Helsinki
Viikinkaari 9, 00790, Helsinki, Finland
jukka-pekka.verta@helsinki.fi

Editor:

Dear Authors,

Your manuscript reports the impact of Vgll3, a transcription factor, on variation of the maturity age in atlantic salmon. The relevance of your study finds unanimous support among all three reviewers. Their careful examination of your manuscript has yielded many constructive comments that will help improve the manuscript. As a result, I ask you to major revise your manuscript before it can be accepted for publication. These revisions will have to address each of the comments made by the reviewers.

RESPONSE:
Thank you for these constructive comments and your attention to our methods. Please see our responses your specific comments below.

a. I particularly believe that the description of effect sizes and their justification must be at the center of your attention in the preparation of the revised manuscript. This concern is shared by two of the reviewers.

   RESPONSE:
   We believe the perspective of our results regarding effect sizes and the biological relevance of small expression changes have now changed with the discovery of alternative isoforms. As you can read from our revised manuscript, we find very good evidence that expression differences we observed in our RT-ddPCR assays were caused by different mixtures of isoforms being expressed in different *vgll3* genotypes. We now believe that because of the alternative isoforms having different 5' UTR sequences and amino-acid sequences, the biological effect of expression differences between the genotypes may be secondary to the effect of alternative protein sequences of *vgll3*.

b. I was personally surprised to see that the quantitative RT PCR you performed did not use control house keeping genes, but were just standardized to the amount of mRNA. This means that variance for the efficiency of cDNA synthesis was not controlled for in your assay.

   RESPONSE:

It is important to note that we used digital-droplet RT-PCR (RT-ddPCR) and not quantitative RT-PCR (qRT-PCR). The two methods share similarities, but are different in crucial aspects. In contrast to qRT-PCR, RT-ddPCR reactions are run to completion (40 cycles) before they are analyzed. Fluorescence from each droplet is then analyzed independently to quantify the number of positive and negative droplet PCR reactions (up to 20 000 individual droplets). Because droplets are analyzed after the completion of PCR, the resulting expression values from RT-ddPCR reactions are much more robust to differences in cDNA synthesis, primer efficiencies, PCR inhibitors etc., as compared to qRT-PCR (Hindson CM, et al. Absolute quantification by droplet digital PCR versus analog real-time PCR. *Nature methods*. 2013;10: 1003–1005. doi:10.1038/nmeth.2633). In RT-ddPCR, inhibition or inefficiency of reverse-transcription is expected to lead to a lower, but still positive, signal amplitude (fluorescence, see e.g. Revision Figure 3 below), not a failed reaction that would falsely be interpreted as no expression.

Our reasoning to use input mRNA amount as data normalization factor follows from the fact that this approach facilitates direct comparison of *vgll3* expression levels between tissues and developmental time points (a major goal in our study). This would not be the case if data were normalized to a housekeeping gene, the expression level of which varies between tissues and developmental time-points, and thus confounds any expression dynamics observed in *vgll3* itself. To illustrate this point, we compared the expression of *vgll3* to two commonly used housekeeping genes, *elf1a* and *gapdh*, between tissues using published RNA-seq data (Lien, S., et al. (2016). The Atlantic salmon genome provides insights into rediploidization. *Nature*, *533*(7602), 200–205. http://doi.org/10.1038/nature17164), that we have re-analyzed (Revision Figure 1). From Revision Figure 1 it is clear that neither *elf1a* nor *gapdh*, both widely-used housekeeping genes, show stable expression across tissues, and would therefore not be suitable for our study.

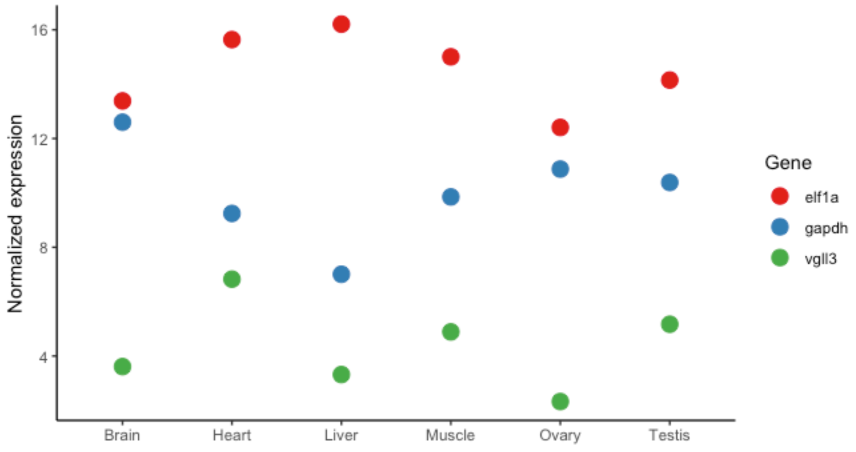

Revision Figure 1. Comparison of vgll3 expression to housekeeping genes.

One further consideration against using housekeeping genes in normalization is the scarcity of established, lowly expressed housekeeping genes. From Revision Figure 1 we can observe that *vgll3* is much more lowly expressed compared to housekeeping genes. In fact, *vgll3* expression is in the lowest 32% quantile of all expressed genes in testes (genes with RPKM > 1, *N*=20537, re-analyzed RNA-seq data as above). From this follows that *vgll3* and most housekeeping genes cannot be analyzed using the same mRNA dilutions because the more highly expressed housekeeping gene would saturate the RT-ddPCR reaction (i.e. all ~ 20 000 droplets per reaction would show signal, in which case abundance cannot be calculated). Using a more highly expressed housekeeping gene for normalization would therefore mean that each sample would need to be diluted to two concentrations, which is prone to accumulating pipetting errors, and to be analyzed using two RT-ddPCR reactions, which would double the cost of the experiment.

Finally, any variation in cDNA synthesis between samples is expected to contribute to excess variation i.e. noise, in the data, rather than leading to false-positive differences between genotypes. In contrast to allocating resources for analysis of housekeeping gene expression, we opted to maximize the number of biological replicates, which should ultimately lead to a more robust biological interpretation of the data.

To further validate our approach of using mRNA amount as a normalizing factor in RT-ddPCR, we performed new experiments comparing mRNA normalization to normalization using a housekeeping gene reported to have stable expression across male puberty,

scamp1 (Kjaerner-Semb, E., et al. (2018). Vgll3 and the Hippo pathway are regulated in Sertoli cells upon entry and during puberty in Atlantic salmon testis, *Scientific Reports*, *8*(1), 1912. http://doi.org/10.1038/s41598-018-20308-1).

We designed and validated a new TaqMan assay for *scamp1,* and re-analyzed samples from Figure 3 in duplicates with normalization to *scamp1* expression level. The results remained unchanged (Revision Figure 2).

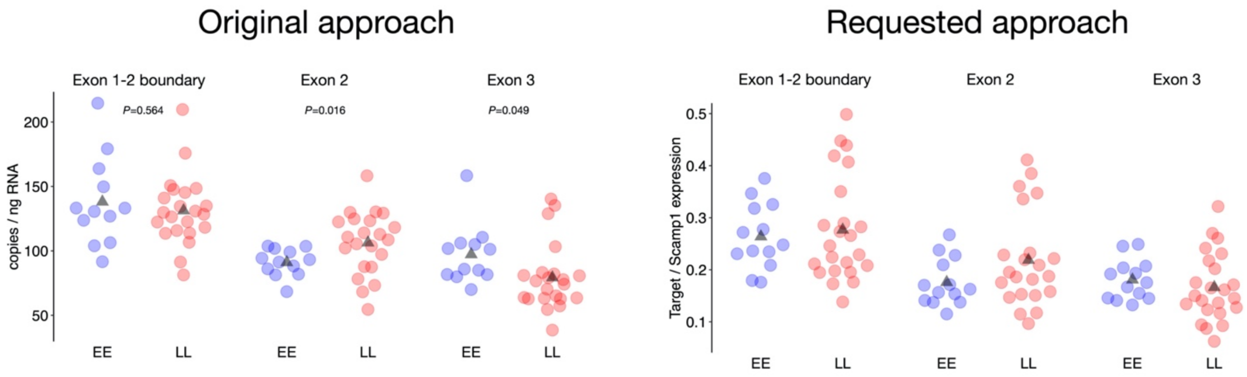

Revision Figure 2. Comparison of results with normalization to input RNA amount versus normalization to a housekeeping gene (Scamp1).

c. I also believe that the magnitude of the cis-regulatory change you report is very small, which requires a careful discussion of both potential technical limitations and putative relevance of tiny changes in cis-regulatory activity.

RESPONSE:
In our revised discussion, we now pay particular attention to the technical limitations of our study as well as clarifying the possible biological relevance of the observed differences. We believe that in light of our revised results, the small expression differences are a proxy for, and may be secondary in effect to, variation in transcript structure.

d. Vgll3 is also highly expressed in the heart and knowledge regarding its potential pleiotropic action should be discussed in more detail.

RESPONSE:
We have broadened the discussion of the possible pleiotropic functions of *vgll3* to include heart tissue. (Lines 452-455)

e. Finally, I would like to ask you to broaden the discussion of your data for the broader readership of our journal. It seems indeed a bit far fetched to claim in the discussion that your study indicates that the evolution of life history is more likely to be regulatory than coding.

RESPONSE:

We have revised the discussion according to our revised results and the reviewer's comments. We now discuss the cross-species generality of isoform-mediated phenotypic differences and the possible underlying molecular mechanisms; the limitations of our study and differences from previous studies; possible biological effect of isoform variation, and regulatory versus coding changes in driving phenotype divergence in evolution.

Reviewer #1:

In this study, the authors investigate how genetic variation in the regulatory sequence of a gene previously identified by GWAS influences a fitness-related trait, the age at sexual maturity in Atlantic salmon males. By crossing males and females with known genotype at the vgll3 locus (a transcription co-factor gene) and raising their progeny in a common environment, they confirmed that allelic variation at this locus was associated with the timing of male puberty. They then quantified vgll3 mRNA levels in ten tissues at different developmental stages, showing that the expression of this gene was elevated in immature testes and decreased after sexual maturation. In addition, allelic variation at the vgll3 locus was associated with a 20% change in mean vgll3 mRNA level in immature testes. By quantifying expression of each vgll3 allele in heterozygote individuals, the authors conclude that the expression difference between the two alleles is largely explained by cis-regulatory variation. They propose a plausible model whereby a cis-regulatory change in a vgll3 enhancer would affect the average expression level of vgll3 in immature testes, contributing to variation in male puberty timing.

By unravelling a molecular mechanism underlying variation in a key life history trait of a non-model organism, I expect this work to have a broad impact on the readership of PLoS Genetics. I particularly appreciated the clarity and concision of the introduction and discussion. However, I have a few concerns that I think should be addressed before publication. In my opinion, the article would benefit from a more detailed description of some of the methods used and from showing more clearly critical controls in some figures, as suggested below. This would strengthen the conclusion that cis- regulatory variation contributes to the expression difference observed between vgll3 alleles.

Here is my list of concerns and suggestions ranked from highest to lowest priority:

1. 1) In page 9, the authors wrote: “the average magnitude of the cis-regulatory differences observed [...] was large enough to account for 62% of the average expression differences between [...] homozygotes.”. I do not understand how they obtained this 62% estimate. They should explain it in the Results and Methods sections, since this is critical to support the conclusion that the expression difference between vgll3 alleles is largely cis-regulatory. I found this 62% estimate surprisingly high since the L allele only represented 53% of total vgll3 mRNA in heterozygotes (as compared to 50% expected in absence of cis-regulatory effect) and the mean vgll3 mRNA difference between homozygotes was 20%.

RESPONSE:

Thank you for pointing this out. Note that the exact expression differences have changed, since we performed additional replication of the RT-ddPCR reactions (mean of duplicates for *vgll3*EE* and *vgll3*LL* genotypes reported). The average 89% difference was estimated by comparing the *E* vs *L* copy number difference in heterozygotes and the difference in alternative homozygotes as follows: On average *EL* individuals expressed 113 *vgll3* mRNA molecules (per ng of input RNA). Of these, on average, 53.5 molecules were expressed from the *E* allele and 60.3 molecules from the *L* allele, giving an average *L* relative abundance of 53% (60.3/(53.5+60.3)*100=53%). From above we can see that average difference between *E* and *L* mRNA counts is 6.8 mRNA molecules (60.3-53.5=6.8). Correspondingly, the average difference between *EE* and *LL* homozygotes is 15 mRNA molecules (106-91=15). The percentage of *EE*-*LL* difference accounted by *E*-*L* *cis*-expression difference is thus (2*6.8)/15*100=88.9%, as *EE* and *LL* each have two of either the *E* or the *L* allele, respectively.

We added these details to the results and methods (also for the assay in exon 3, lines 326-330, lines 336-338 and lines 648-658).

2. 2) Related to the first point, in the introduction the authors proposed the hypothesis that a cis- regulatory change can underlie the association between vgll3 genotype and age at maturity since the strongest association (in the previous GWAS study) mapped to a non-coding sequence 7000 bp upstream of vgll3 coding sequence. While this seems to be the most likely explanation, previous studies in other organisms showed that genetic variation in an enhancer can have trans-regulatory effect on expression of nearby genes (Tian et al. PLoS Genet. 2019 doi: 10.1371/journal.pgen.1008152; Bateman et al. Genetics 2012 doi: 10.1534/genetics.112.140954). The authors could mention this alternative hypothesis in the introduction or in the discussion.

   RESPONSE:

   This is a good point, thank you. We added this alternative hypothesis to the introduction (lines 98-101).

3. 3) The null hypothesis for testing allele specific expression in Figure 3B was obtained from heterozygote DNA. This is an essential control to assess the statistical significance of the cis- regulatory effect observed, and I am glad the authors did it. However, they do not provide much information about this control. How many replicate samples were used

to obtain the dashed line on Figure 3B? How were these data used to compute the P-values shown on Figure 3B? I would also suggest mentioning in the main text why this control was performed, which I suppose was to account for biases in the specificity of the PCR primers. This would more convincingly convey that the 53% deviation (weak but significant) toward the L allele is not an experimental artifact.

RESPONSE:

Thank you for raising this point. Our experiments included heterozygous DNA as control for each RT-ddPCR run we performed. The *N* for these controls is 10. As you point out, the reason to use a DNA control is to account for any differences in the efficiencies of the TaqMan assays for each allele. In practice the abundances of the two alleles in our DNA control were extremely similar (*L/(L+E)*100*=50.44% for exon 2 and 49.4% for exon 3, respectively), indicating that the assays were performing with nearly equal efficiency for both alleles. The assays for exon 1-2 and the alternative 5' UTR both spanned intron-exon boundaries, and therefore we could not use genomic DNA as control (the assay does not amplify with DNA as template). We assumed 50% efficiency for these assays. We point out that even without DNA control, our analysis of an independent RNA-seq data set supports our results of *cis*-regulatory expression difference in the alternative 5' UTR.

Computation of the *P*-values for the data in Fig 3B follows from the Poisson nature of the ddPCR count data (see e.g. Hindson CM, et al. Absolute quantification by droplet digital PCR versus analog real-time PCR. *Nature methods*. 2013;10: 1003–1005. doi:10.1038/nmeth.2633). For each channel (allele), we know the number of positive droplets as well as the number of negative droplets. Testing for differences in the abundance of the alleles can therefore be performed by testing for difference in the Poisson rate of positive droplets over all droplets (i.e. rate-ratio test). In the statistical test, the DNA control is used as the null rate ratio (the *r* parameter in *R* function *poisson.test()*).

We revised the results section and the methods to make these points clearer (Lines 323-324, lines 636-646). Note that the data from heterozygotes is now presented in Figure 5.

4. 4) For a non-specialist in fish biology, the methods used to assess sexual maturity seem a bit subjective (Page 13: "We [...] assessed the maturity status of males by manually

testing for the presence of running milt"; Page 14: "We classified each male fish visually as immature (not enlarged, transparent color gonads), pubertal (slightly enlarged, transparent or white color gonads) or mature (enlarged white color gonads)"). I am wondering if it would be possible to collect more quantitative data about this central phenotype, for instance by weighing the dissected gonads. At the very least, it would be helpful for non-specialist readers to mention in the text that the age of sexual maturity can be difficult to measure accurately. I guess this is why the authors used quantitative measures such as amh/igf3 ratio, which could be stated more clearly.

RESPONSE:

We revised the methods section for clarity in assessing sexual maturity (Lines 523-524). Sexual maturity checks were performed twice for two different purposes. Page 13 referred to maturation assessment of live fish that was performed for all fish at the start of the breeding season to model maturation probabilities. This assessment needed to be non-lethal as continuation of the experiment after a lethal maturation assessment would have been impossible. Testing for running milt is the standard procedure in fish husbandry (academic and non-academic). We emphasize that this does not affect the samples on which molecular analyses were conducted (see below).

Page 14 referred to fish sacrificed for molecular sampling along the experiment. For sacrificed animals we could visually inspect the gonads. You are correct in that the percentage of gonad weight of the total body weight (i.e. gonadal-somatic index) is a commonly used metric to quantify sexual maturity in fish. In our case this metric was not useful however, as the immature gonads had no weight measurable with a standard scale with detection limit of 10 mg. The gonadal-somatic index in our case was therefore reduced to a binary factor of "no measurable weight" versus the typical ~10% of body weight of the mature individuals. You are correct in that we used *amh/igf3* expression ratio to validate our visual classifications (Fig S5). We took notice of your comment and state that our visual classifications of gonad morphologies were consistent with molecular classification based on *amh/igf3* ratio (Lines 190-193). We also included discussion of uncertainty in non-lethal assessment of sexual maturity as possible source of error/variation (Line 476).

5. 5) In page 5, the authors mentioned a 9% vs 0% difference in mean maturation probability and a 10 times higher frequency of maturation in individuals with vgll3*EE

genotypes compared to individuals with vgll3*LL genotypes. I do not understand the distinction between “mean maturation probability” and “frequency of maturation”, and how these values were obtained. Related to this point, it is unclear how the error bars were calculated on Figure 1A. It would also be nice to mention the number of individuals in the legend. For clarity, the legend of Figure 1B should mention the type of model used (generalized linear mixed model).

RESPONSE:

Thank you for pointing this out. Maturation *probability* is the estimate we modeled based on the observed *frequencies* of mature versus non-mature fish at nine months post-hatching. We think both probabilities and frequencies are of interest to the readers, as maturation probability can be thought as a more generalizable metric across experiments, whereas maturation frequencies always depend directly on the experimental setup. Reporting maturation probability is however non-trivial, as the genotype-phenotype relationship needs to be modeled on a probit-scale (because of the binary mature versus non-mature response variable). This means that to be truly honest to the data, we would need to report *P*-values, credible intervals and maturation probabilities on the probit-scale. We do this for the *P*-values and credible intervals, but we think it would not be digestable for maturation probabilities. To help the reader interpret the results, we have opted to transform the probit estimates for maturation probability and credible intervals back to “normal” probability scale, to which the 0% versus 9% mean maturation probability and Fig 1A referred to. Please also note that we have revised the method of estimating the probabilities of maturation from the probit-model (more details in the following preprint: https://www.biorxiv.org/content/10.1101/780437v4), and that our new estimates for the average probabilities are now 20.7%, 15.4% and 3% for *vgll3*EE, vgll3*EL* and *vgll3*LL* genotypes, respectively. We revised the section for clarity (Lines 127-136).

The error bars are confidence intervals from our statistical model as per the Methods section: “*We estimated maturation probability using a generalized linear animal model with probit-link function under Bayesian Markov Chain Monte Carlo simulations with the R-software version 3.6.1 and package MCMCglmm for the following model: y = vgll3 + length + a + t +e , where y is a vector of maturation binaries, length is mean-centered and variance scaled natural logarithm of length (cm), vgll3 is the genotype, a is the additive genetic relationship-matrix predicted animal effect, t is the common environmental tank effect and e the error term (with fixed variance of 1). We used priors following a χ_1^2*

distribution and based inferences on 10,000 samples collected every 1,000 iteration after burn-in of 10,000." We added a reference of the model to the figure legend.

6. 6) The experimental design could be explained with a bit more details to be more accessible for non-specialists. For instance, I suggest to move Figure S1 as a panel in Figure 1. If I understood well, each of 16 males were crossed to 2 females and each of 16 females were crossed to 2 males to produce a total of 32 families. Were they all crossed simultaneously or consecutively? How were the families with common parents distinguished? The authors wrote on Page 13 that each tank included on average six individuals per family, but it is unclear whether each tank included individuals from all families or not. Also, I assume that a given number of individuals from each family was randomly selected for each experiment, but this process is not clearly explained. For instance, did the experimenters know the vgll3 genotype when assessing maturity or was it done in a blind way?

   RESPONSE:
   Thank you for pointing out that these details were unclear. We revised the methods section to clarify these issues (Lines 489-493). We hope this is adequate clarify the experimental design without the need to add figure S1 as a panel to Figure 1.

7. 7) In Figure 2, all expression measures were obtained using a digital PCR approach. Since this approach is more sensitive than standard qPCR, the authors should explicitly mention that they used this approach in the main text as well as in Figure 2 legend. They mentioned in the Methods that they verified the specificity of all TaqMan assays with genomic and synthetic DNA templates, but did not provide data for these important controls. I suggest that they include a supplementary figure showing the specificity of the primers used in digital PCR assays.

   RESPONSE:
   We added a reference to RT-ddPCR to the results section and the figure legend (Lines 167-169). See Supplemental figure 10 for RT-ddPCR controls of *vgll3* TaqMan assay (also Revision Figure 3 below).

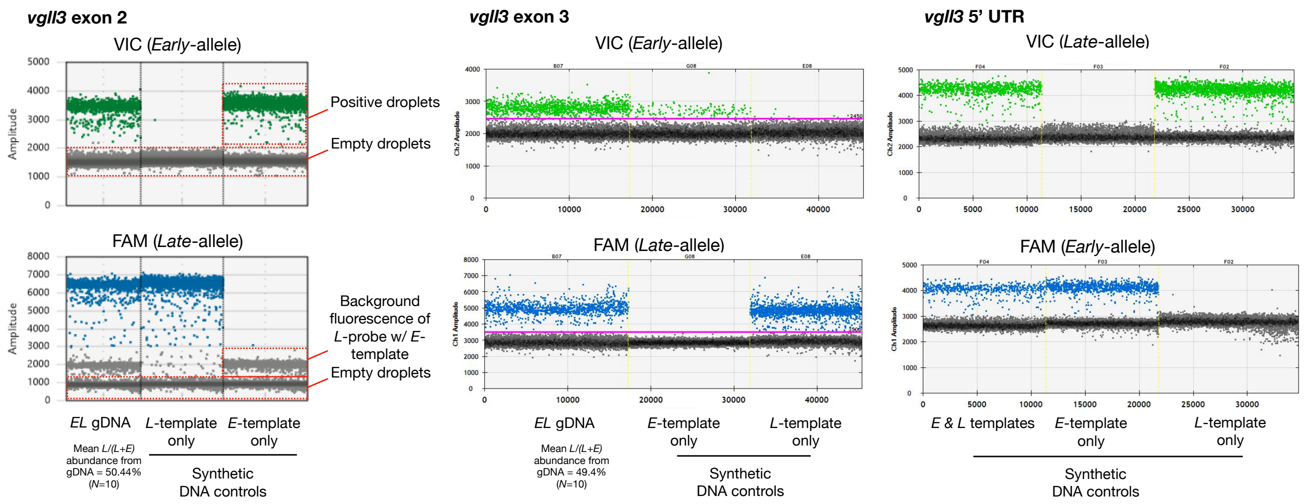

Revision Figure 3. Genomic DNA and synthetic DNA controls of vgll3 TaqMan assay.

8. 8) In Figure 3B, the y-axis should be L / (L + E) instead of L / E. The legend mentions black and grey colors, while the color scale on the figure is dark to light green. I also suggest using different symbols to distinguish data points with P-value < 0.05 (triangles for instance).

RESPONSE:

Thank you for pointing this out, we have changed the y-axis label and legend accordingly.

9. 9) In Page 3, the authors wrote: “the transcription co-factor gene vestigial-like 3 (vgll3) was recently found to explain nearly 40% of variation in sea-age at maturity”. Was this association found only in males, also in females, or regardless of sex? I think this information should be mentioned in the text.

RESPONSE:

We added these details to the text (Line 82).

10. 10) There is a grammatical problem with the first sentence of the third paragraph in Page 13: “Before first feeding and transfer...”.

RESPONSE:

We revised the sentence (Lines 502-505).

Reviewer #2:

The paper by Verta et al. addresses the molecular basis of life history differences in Atlantic Salmon. From prior work, they identify a candidate transcription factor, vgll3, that is associated with age at maturity and confirm this association using crosses in the laboratory. A number of factors suggest that changes in vgll3 expression may be important and the authors analyze expression across multiple tissues and time points, identifying a change in vgll3 expression in the testis upon maturity. They then test for regulatory differences among individuals with different vgll3 'alleles' finding a small, but significant cis-regulatory difference in expression prior to maturity. Overall, the paper is clearly written and the authors generally are careful and precise in their claims and interpretations of the data. I do, however, have a few concerns with the work, one experimental, one interpretive, and the other about the presentation, that should be addressed.

1. There appears to be a gap in the experiments with respect to the LL genotype. Time series data for vgll3 expression in a number of tissues was done for the EE genotype. This includes multiple immature and mature fish. In addition, for both the EE and LL genotypes, vgll3 expression was measured and compared in immature fish. However, there is no corresponding data on vgll3 expression levels in mature LL fish. As such, the data show that in EE fish, vgll3 expression drops upon maturity and that prior to maturity, vgll3 expression is higher in LL fish. It doesn't show what happens upon maturity in LL fish. It seems like the authors assume that vgll3 expression also drops upon maturity in LL fish, but this claim is not tested as far as I can tell.

RESPONSE:
It seems that our sentence "*In order to investigate the functional mechanisms resulting in earlier maturation in males carrying the vgll3*EE genotype and to establish the tissues and temporal time-points of relevance for vgll3 expression…*" has been mis-leading in this regard. *Vgll3* genotypes were sampled in equal numbers and *vgll3*EE* and *vgll3*LL* genotypes were analyzed in roughly equal numbers (differences due to failed samples). The data presented in Figure 2 are indeed summarized from both *vgll3*EE* and *vgll3*LL* genotypes. In addition, immature ovary, immature testes and mature testes expression was measured from all *vgll3* genotypes (see Revision Figure 4 below). We re-phrased the relevant sentences to make this clearer (Lines 163-169) and colored Figure S3 by genotype.

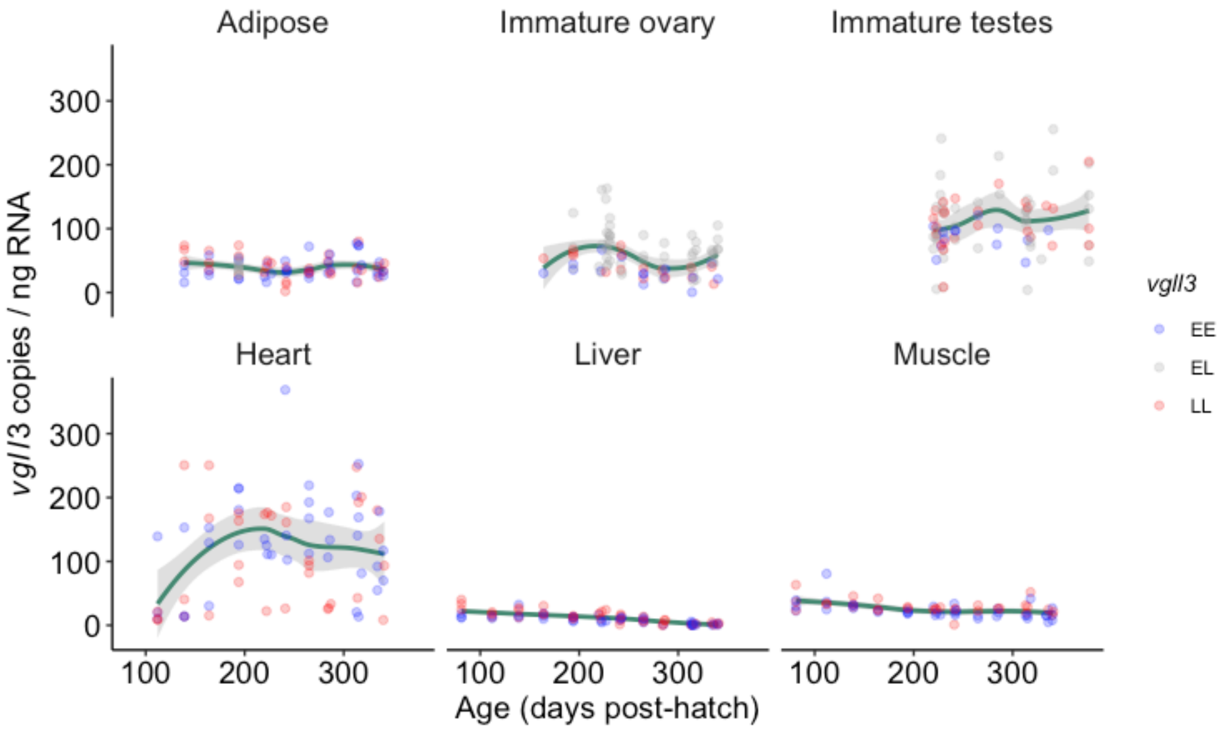

Revision Figure 4. Vgll3 expression in tissues - colored for genotype.

You are correct in that *vgll3*EE* genotypes are over-represented in the individuals analyzed for *vgll3* expression in mature testes. This is due to the effect of vgll3 genotype on maturation probability, which results in a low frequency of maturation in individuals with *vgll3*LL* genotypes. We succeeded to obtain mature testes expression from one *vgll3*LL* individual, which shows equally low expression compared to *vgll3* EE* individuals (Revision Figure 5). Our revised Supplemental Figure S3 includes mature testes and now indicates genotype information.

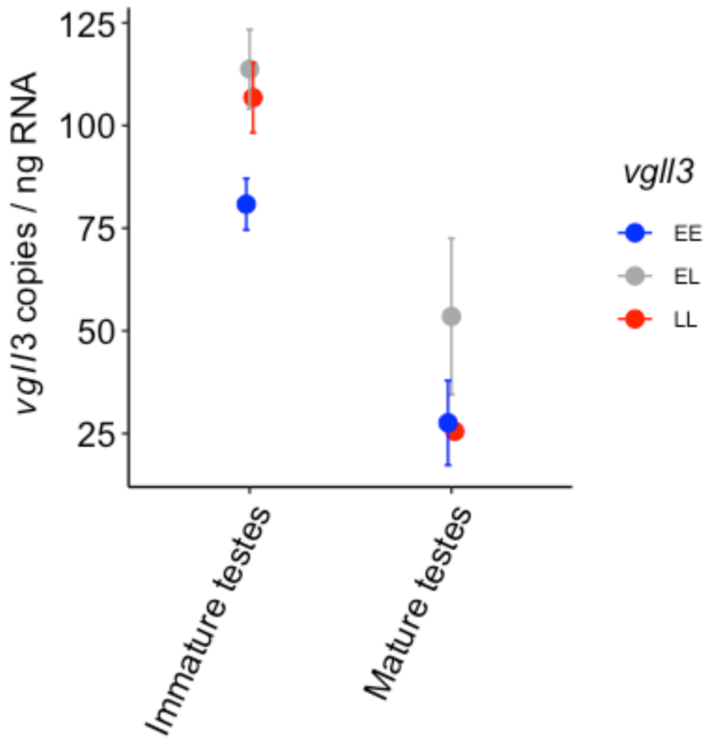

Revision Figure 5. Vgll3 expression in immature and mature testes - colored by genotype.

2. The EE and LL genotypes do a good job of explaining variation in time to maturity and the cis-regulatory difference measured between these alleles accounts for much of the expression difference. However, the EE and LL genotypes do a poor job of explaining variation in expression. Given that the proposed model is that differences in genotype affect expression (in cis) and this in turn affects maturity, this lack of explanatory power at the molecular step should be acknowledged. This is not to say that anything is wrong, as finding a molecular association of any kind in such a study is already promising, but I believe it requires discussion as to the possible causes. Some possible causes could be experimental error (i.e. noisy assay), incorrect timing of measurement, or a different molecular step being the key.

RESPONSE:

We agree that it is clear from our Figure 3 that the observed ranges of *vgll3* expression in all genotypes are largely overlapping. We believe that the perspective on the observed small expression differences has now changed with our discovery of alternative transcript isoforms. Our revised results suggest that expression differences reflect differences in isoform expression between *vgll3* alleles, such that the effect of *vgll3* expression would be secondary to differences in protein structure (Lines 285-288).

Additionally, we would like to note that have shown that *vgll3* genotype associates with *the likelihood of reaching maturity*, not with the presence or absence of a trait (explained in more detail in the following pre-print: https://www.biorxiv.org/content/10.1101/780437v4). What we propose to explain this association is a liability model, where carrying a certain *vgll3* allele influences the probability of reaching early maturation through changing the tendency of expression in *vgll3* isoforms. In human disease genetics terms, *vgll3* would be considered as an important "risk factor" for reaching early maturation, not determining maturation age directly. To assume that carrying a certain *vgll3* genotype would directly translate to a maturation phenotype as a Mendelian trait is therefore not something we propose.

We acknowledge that this is an important point of discussion and we have revised the discussion to better acknowledge the variation around mean expression levels, as well as the possible sources (Lines 427-442).

3. Framing of the proposed model needs work. In the paper, it is presented in the discussion as the data 'supporting' this model. I read this as indicating that the model had previously been establish. However, no reference was given. Alternatively, if this is a new model, phrasing such as the data 'suggesting' a model would be more consistent.

In addition, this section contains new analysis and introduces a number of additional molecular steps (e.g. Teads and Yap). This would seem to fit better in the results section of the paper.

Finally, a negative correlation between vgll3 expression and yap1 expression is indicated in the text, but figure S11 doesn't show this result. Instead S11 shows a negative relationship between vgll3 expression and maturity in a new data set (which is consistent with the rest of the paper and a nice confirmatory step) and a positive relationship between yap1 and maturity. This later relationship seems to be the basis of the new claim by the authors, but it is simply a correlation. While a useful insight that warrants follow-up, it doesn't rise to the level of an established claim about the molecular relationship between vgll3 and yap1.

RESPONSE:
We changed "supporting" to "suggesting" as per your comment. Please note that we have revised the proposed model completely, and propose that the genetic control of the maturation phenotype is alike to the mechanism of sex determination in many insects, which is mediated through the expression of alternative isoforms of a transcription factor gene. We also revised

the discussion of the model to reflect that it serves as a hypothesis, as well as the need for follow-up studies as you noted (Lines 388-390, lines 415-416).

Minor

1. While the main text is precise with respect to the experiments, causation is overstated in the abstract. Unless I'm mistaken, inbred lines were not used and individual fish vary at more than just the vgll3 locus. Thus the words mediate and control in the abstract are stronger than what is shown in the paper, which are correlations.

RESPONSE:
We re-phrased the abstract to reflect these valid points (Lines 41-43).

2. The liability model is overstated in the author summary. It is stated as a fact instead of as a hypothesis that arises from the current work.

RESPONSE:
We re-phrased the author summary (Lines 50-57).

3. Figure 2A. Muscle and immature ovary are shown but not mentioned in main text. Mature testes is only shown in supplement even though it is focal comparison of the paper. Likewise, stripped testes and ejaculate are shown in the following figure, but the time series data is relegated to the supplement.

RESPONSE:
We added reference to immature ovary and muscle to the text (Lines 174-175). We added the mature testes, stripped testes and ejaculate data to panel A of Figure 2.

Pg. 8. And Figure 3A. Only the difference in vgll3 RNA counts is reported in the text between homozygous genotypes. It would be helpful to have the average values as well for each genotype. In addition, the value reported for heterozygotes in the test (27) doesn't match the figure.

RESPONSE:

The value reported for heterozygotes (27) referred to in the original text is the difference between EL and EE means (and the values in brackets the 95% confidence interval). Note that we have completely revised Figure 3 that now includes only homozygote expression data.

Figures S8 and S9. To support the claims in the text, p-values showing no difference between EE and LL genotypes are needed. Also, need trend lines on figures to see change with age (or not).

RESPONSE:
We added these details to the figures and figure legends (Note: now figures S7 and S8).

Figure 3B. y-axis label is confusing. From the text, I believe this should be something like '% L allele' of 'L allele abundance (%)'.

RESPONSE:
Thank you for pointing out this typo. We revised the y-label.

Figure S11. Need SD or SE bars. Or given, that it is only three data points, need to show all of them to get a sense of the expression variation within and between groups.

RESPONSE:
We revised Figure S11. Please note that this data is now presented in Figure S6. Unfortunately, the *Clust*- normalization method we used (*Clust: automatic extraction of optimal co-expressed gene clusters from gene expression data. (2018), Genome Biology, 19(1), 172. http://doi.org/10.1186/s13059-018-1536-8*) does not provide SD or SE for gene expression pattern estimates. To accommodate your suggestion, we added a second panel with *DESeq2*-normalized expression values for the replicates. Note, that patterns from the two methods are not comparable, since the idea behind using *Clust* is to be able to compare gene expression patterns independently from their mean expression levels, whereas *DESeq2* normalization does not correct for means.

Reviewer #3:

Verta, Primmer, et al present an exceptional study that links cis-regulation of the vestigial-like 3 (vgll3) to alternative life-history traits in Atlantic salmon. They use a series of experiments of seeming monumental effort - 32 familes phenotyped at multiple time points, gene expression measured at 13 time points in 10 tissues – and clever gene expression analyses and visualizations to link expression variation with growth and maturation phenotypes. They show that the early maturation phenotype is associated with downregulation of vgll3 through cis-regulatory variation. The manuscript unfolds nicely with a series of clearly articulated hypotheses and their tests. I think the study and manuscript are exceptional and meet the effort-level and interest/scope typical of high-profile PLoS Genetics papers. I recommend it be published with consideration of the minor points/questions I bring up below.

Minor points:

1. Abstract – I think "variation in" should be included in the first sentence, e.g., "how evolution shapes variation in life histories." Minor point of preference.

RESPONSE:
Thank you for this suggestion. We revised the abstract accordingly (Lines 30-31).

2. I don't know that the first part of the author summary section is clear enough for all readers to get behind. I think it should be a statement that everyone knows or agrees with. Is "much of the world's intraspecific biodiversity" "due to alternative strategies to reach reproduction"?

RESPONSE:
Thank you for making this point. We agree, and have revised the author summary from a different point of view (Lines 50-51).

3. Age at maturation as a complex trait – In a previous study cited in wild populations (ref. 4), vgll3 explained ~40% of the variation in sea-age at maturity. It seems like less variation is explained in this controlled setting, surprisingly. I would suggest this be addressed in the discussion.

RESPONSE:

Thank you for bringing up this discussion point. We address the possible technical and biological causes of differences in genotype-phenotype associations between the two studies in the revised discussion (Lines 470-478). We would like to note that we think the analytical and experimental differences between the studies are the main source underlying the seemingly lower proportion of variation explained in our study.

4. Figure 3B. Shouldn't the y-axis be "L/E+L allele abundance %"? Not a ratio, but a proportion?

RESPONSE:
Thank you for pointing this out. We have corrected the typo.

5. Discussion – Excellent discussion, including of cis vs. coding variation as important in life-history trait evolution. It is beyond the scope of this study, but I wonder if it would warrant mention that functional validation, as in manipulation of allele-specific expression, would be an important future direction. To this point, I would not include "functional validation" as a keyword of the study.

RESPONSE:
Excellent point, we revised the discussion (Lines 440-442) and deleted the keyword.
