# Supplementary material for: Cis-regulatory differences in isoform expression associate with life history strategy variation in Atlantic salmon
Source: PLoS Genet. 2020 Sep 30;16(9):e1009055. doi: 10.1371/journal.pgen.1009055 (PMC7549781; doi:10.1371/journal.pgen.1009055)
Supplement: Attachment — Submitted filename: Review_revision.pdf [file pgen.1009055.s014.pdf]

In this revised version of their manuscript, Verta *et al.* addressed the different issues raised by the reviewers and went further by characterizing a new isoform of the *vgll3* transcript and by showing that the expression of this new isoform was affected by *cis*-regulatory variation existing between the two alleles of *vgll3*. This substantial amount of additional work, carefully performed, led them to propose a new model in the discussion whereby the association between *vgll3* genotype and age at maturity was driven by *cis*-regulatory variation in the expression of different *vgll3* transcript isoforms.

I particularly appreciated the detailed and well-argued responses of the authors to the reviewers and editor's comments. Regarding an issue I had with the initial submission, I now find the methods to be much more clearly explained with appropriate amount of details. The existence of *cis*-regulatory differences affecting the expression of *vgll3* transcript isoforms, which is the main finding of the study, is convincingly demonstrated thanks to the large number of samples and appropriate statistical analyses. The high sensitivity of the experimental approach was essential due to the small magnitude of the *cis*-regulatory differences reported.

Although I really appreciated how the authors acknowledged the limitations of their study in the discussion, I think their main conclusion is still overstated in some sentences that need to be nuanced (or phrased differently) before publication. This is the case for instance in the legend of Figure 6 or in this sentence at line 383: "We found strong evidence that *vgll3* genotypes associated with maturation age phenotypes do so through a mechanism of *cis*-regulated differences in transcript isoform expression in immature testes." To me, this study describes a novel *cis*-regulatory mechanism that could potentially contribute to the association between *vgll3* genotype and maturation age; however, it does not provide any direct (functional) evidence that the *cis*-regulatory differences are indeed involved in maturation age. As mentioned in the discussion, the association with maturation age could also be explained by missense SNPs in *vgll3* coding sequence. In addition, given the small magnitude of the *cis*-regulatory differences detected (even when considering only the newly discovered transcript isoform), the functional consequences of these *cis*-regulatory changes are not obvious.

With more nuanced conclusions, I would recommend publication of this study in *PLoS Genetics* given the remarkable amount of careful work and the convincing discovery of *cis*-regulatory variation affecting the expression of different *vgll3* transcript isoforms.

I also have the following minor comments:

1) The text on Figure 4 is too small.
2) The distribution of data points seems bimodal on Figure 5A for the new isoform, suggesting a potential interaction between the *cis*-regulatory change and a *trans*-regulatory factor. The biomodality on Figure 5A could be referenced in the discussion when the authors mention the possibility of *cis*- and *trans*-acting factors (line 430).
3) The opposite direction of *cis*-regulatory difference between exon 2 and exon 3 is very intriguing and not discussed. Could this be explained by another transcript isoform not yet discovered (with splicing that would skip exon 2 for instance)?
