# Supplementary material for: Cis-regulatory differences in isoform expression associate with life history strategy variation in Atlantic salmon
Source: PLoS Genet. 2020 Sep 30;16(9):e1009055. doi: 10.1371/journal.pgen.1009055 (PMC7549781; doi:10.1371/journal.pgen.1009055)
Supplement: Attachment — Submitted filename: verta_et_al_response_to_reviews2.pdf [file pgen.1009055.s015.pdf]

UNIVERSITY OF HELSINKI
FACULTY OF BIOLOGICAL AND ENVIRONMENTAL SCIENCES

Dear Dr. Juliette de Meaux, dear reviewers,

Thank you for your highly beneficial and insightful feedback on our manuscript. We believe the study is significantly better because of your common efforts to evaluate our results and presentation. This peer-review has been a positive experience for us and we appreciate your understanding regarding the delays due to the current situation.

Below you will find our point by point responses to the comments from reviewers. We have now deposited the data, the code, as well as the output from *R* on Dryad (private until acceptance of the manuscript, but can be accessed using the following link: https://datadryad.org/stash/share/ati2k5nMIKGL6d2o-teqNX1UfdPHp1ibDWKHN30NdZ8). We will make the data set public after acceptance and update the accession number in the manuscript.

Sincerely,

Jukka-Pekka Verta
Post-doctoral researcher
Organismal and Evolutionary Biology Research Programme
University of Helsinki
Viikinkaari 9, 00790, Helsinki, Finland
jukka-pekka.verta@helsinki.fi

Reviewer #1:

In this revised version of their manuscript, Verta et al. addressed the different issues raised by the reviewers and went further by characterizing a new isoform of the vgll3 transcript and by showing that the expression of this new isoform was affected by cis-regulatory variation existing between the two alleles of vgll3. This substantial amount of additional work, carefully performed, led them to propose a new model in the discussion whereby the association between vgll3 genotype and age at maturity was driven by cis-regulatory variation in the expression of different vgll3 transcript isoforms.

I particularly appreciated the detailed and well-argued responses of the authors to the reviewers and editor's comments. Regarding an issue I had with the initial submission, I now find the methods to be much more clearly explained with appropriate amount of details. The existence of cis-regulatory differences affecting the expression of vgll3 transcript isoforms, which is the main finding of the study, is convincingly demonstrated thanks to the large number of samples and appropriate statistical analyses. The high sensitivity of the experimental approach was essential due to the small magnitude of the cis-regulatory differences reported.

Although I really appreciated how the authors acknowledged the limitations of their study in the discussion, I think their main conclusion is still overstated in some sentences that need to be nuanced (or phrased differently) before publication. This is the case for instance in the legend of Figure 6 or in this sentence at line 383: "We found strong evidence that vgll3 genotypes associated with maturation age phenotypes do so through a mechanism of cis-regulated differences in transcript isoform expression in immature testes." To me, this study describes a novel cis-regulatory mechanism that could potentially contribute to the association between vgll3 genotype and maturation age; however, it does not provide any direct (functional) evidence that the cis-regulatory differences are indeed involved in maturation age. As mentioned in the discussion, the association with maturation age could also be explained by missense SNPs in vgll3 coding sequence. In addition, given the small magnitude of the cis-regulatory differences detected (even when considering only the newly discovered transcript isoform), the functional consequences of these cis-regulatory changes are not obvious.

With more nuanced conclusions, I would recommend publication of this study in PLoS Genetics given the remarkable amount of careful work and the convincing discovery of cis-regulatory variation affecting the expression of different vgll3 transcript isoforms.

RESPONSE:

Thank you for these comments. We agree that what we have shown are genotype-phenotype associations, not causal relationships, and have revised the manuscript accordingly:

We changed the last sentence of the abstract (lines 46-47) to the following (changes underlined): "These results exemplify the potential importance of regulatory differences as a mechanism for the evolution of life history traits."

We changed the last sentence of the introduction to the following (changes underlined): "Our results reveal a potential functional mechanism that links allele-specific isoform expression differences in vgll3 to variation in maturation timing and thus may mediate variation in life history strategies."

We revised Figure 6 legend to the following (changes underlined): "We hypothesize that cis-regulatory differences influencing vgll3 isoform expression are an important mechanism

contributing to the association between vgll3 genotype and maturation timing. The vgll3*L allele tends express more of the shorter isoform compared to the vgll3*E allele, thus potentially changing the balance between vgll3-controlled differentiation versus self-renewal. TEAD interaction with vgll3 isoforms may tip the balance between tendency for self-renewal (higher expression of the short isoform) and differentiation (long isoform)."

We revised the sentence on line 388 to the following (changes underlined): "Our results provide evidence to suggest that vgll3 genotypes associated with maturation age phenotypes may do so through a mechanism of cis-regulated differences in transcript isoform expression in immature testes."

I also have the following minor comments:

1) The text on Figure 4 is too small.

RESPONSE:

We increased the font on Figure 4 labels.

2) The distribution of data points seems bimodal on Figure 5A for the new isoform, suggesting a potential interaction between the cis-regulatory change and a trans-regulatory factor. The biomodality on Figure 5A could be referenced in the discussion when the authors mention the possibility of cis- and trans-acting factors (line 430).

RESPONSE:
This is a good point and thank you for raising it. We have revised the discussion to include this possibility (lines 439-441): "Bimodality in the expression of vgll3 5'UTR (Fig 5A) suggests that the influence of as yet unknown trans acting factors on vgll3 isoform expression cannot be ruled out."

3) The opposite direction of cis-regulatory difference between exon 2 and exon 3 is very intriguing and not discussed. Could this be explained by another transcript isoform not yet discovered (with splicing that would skip exon 2 for instance)?

RESPONSE:
This is indeed a possibility that we think should be addressed with alternative approaches in the future (e.g. long-read RNA-seq). To address this possibility, we added the following sentence to the discussion (lines 396-398): "Additionally, the opposite direction of association between vgll3 genotype and cis-regulatory expression in exons 2 and 3 indicates the possible existence of additional transcript isoforms."

Reviewer #2:

The authors have addressed all of my initial concerns. The revised model is presented clearly and provides a precise hypothesis for future work. Overall the paper is important, well written, and will make an excellent contribution to the field.

RESPONSE:
Thank you for these encouraging comments and we highly appreciate your comments on our previous version.

Reviewer #3:

The study by Verta et al provides an exceptional example of genotype-phenotype association for a critical life-history trait, age at maturity in Atlantic salmon, a species of great economic interest. The authors thoroughly revised their study according to reviewers and editor suggestions and performed new experiments that changed their results and model, but did not change their conclusions and the significance of their study. The authors sufficiently addressed my questions, comments, and concerns in their revised manuscript. They revised the mechanism linking age at maturity to isoform/splice variation of vgll3 rather than allele specific expression adding new data and a revised model. I think this revised article would make an important contribution of interest to the broad readership of PLoS Genetics.

RESPONSE:
Thank you for your these very positive comments, as well as for your helpful comments on our previous version.
